# Supplementary material for: Rotational and nuclear-spin level dependent photodissociation dynamics of H2S
Source: Nat Commun. 2021 Jul 22;12:4459. doi: 10.1038/s41467-021-24782-6 (PMC8298612; doi:10.1038/s41467-021-24782-6)
Supplement: Supplementary file 1 — Supplementary Information [file 41467_2021_24782_MOESM1_ESM.pdf]

Supplementary Information

Rotational and Nuclear-Spin Level Dependent Photodissociation Dynamics of H_2S

Zhao et al

Supplementary Table 1. (a) Experimental wavenumbers and wavelengths of transitions contributing to the four $^1B_1 – \tilde{X}\ ^1A_1$ features of interest, along with the corresponding excited state $\langle J_q^2 \rangle$ values, where $q = a, b, c$ refer to the inertial axes (*a* is parallel to H……H, *b* bisects the angle ∠H–S–H, and *c* is perpendicular to the molecular plane). (b) Relative populations of the various $\tilde{X}$ state rotational levels, with energies *E*, assuming a nuclear spin (T_{ns}) temperature of 300 K and rotational temperatures (T_{rot}) of 3 and 15 K. These values were determined using PGOPHER [1] and the previously reported [2] spectroscopic parameters for the $\tilde{X}\ ^1A_1$ and $^1B_1$ states, but with the band origin value reduced by 0.2 cm^{-1} (to 71897.1 cm^{-1}) and excited state centrifugal distortion constants as for the ground state cation.[3] Transitions involving *ortho*- / *para*- nuclear spin levels of H_2S are displayed with **bold**/normal font, respectively. Note that the line intensities in any measured PHOFEX spectrum depend on the ground state level populations, the transition linestrengths and the various excited level dependent decay rates and product branching ratios.

(a)

Transition wavenumber / cm^{-1}	Transition wavelength / nm	$^1B_1$ state, J_{KaKc}'	$\tilde{X}\ ^1A_1$ state, J_{KaKc}''	$^1B_1$ state		
				$\langle J_a^2 \rangle$	$\langle J_b^2 \rangle$	$\langle J_c^2 \rangle$
**71877.7**	**139.125**	$\mathbf{0_{00}}$	$\mathbf{1_{10}}$	**0**	**0**	**0**
71895.5	139.091	1_{01}	1_{11}	0	1	1
**71896.3**	**139.089**	$\mathbf{2_{02}}$	$\mathbf{2_{12}}$	**0.59**	**1.48**	**3.93**
71896.9	139.088	2_{12}	2_{02}	1	1	4
**71898.4**	**139.085**	$\mathbf{1_{11}}$	$\mathbf{1_{01}}$	**1**	**0**	**1**
71916.5	139.050	1_{10}	0_{00}	1	1	0
**71934.1**	**139.016**	$\mathbf{2_{11}}$	$\mathbf{1_{01}}$	**1**	**4**	**1**
**71936.2**	**139.012**	$\mathbf{2_{20}}$	$\mathbf{1_{10}}$	**3.41**	**2.52**	**0.07**
71937.4	139.010	2_{21}	1_{11}	4	1	1

(b)

J_{KaKc}''	E / cm^{-1}	Relative populations	
		T_{rot} = 3 K	T_{rot} = 15 K
0_{00}	0	0.249	0.132
$\mathbf{1_{01}}$	**13.76**	**0.702**	**0.420**
1_{11}	15.09	0.001	0.093
$\mathbf{1_{10}}$	**19.37**	**0.047**	**0.245**
2_{02}	38.07		0.017
$\mathbf{2_{12}}$	**38.30**		**0.067**
2_{11}	51.14		0.005
$\mathbf{2_{21}}$	**55.16**		**0.013**
2_{20}	58.32		0.002

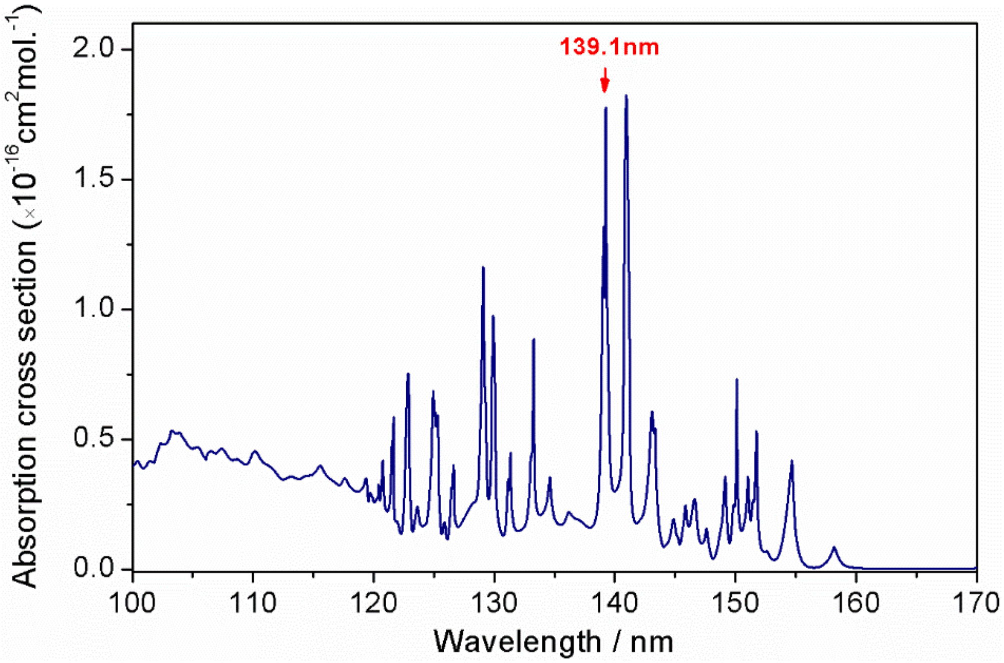

Supplementary Figure 1. Absorption Spectrum of H_2S. Electronic absorption spectrum of a room temperature, gas phase H_2S sample in the wavelength range 100-170 nm, with the 139.1 nm band of current interest highlighted. (Adapted from ref. 4)

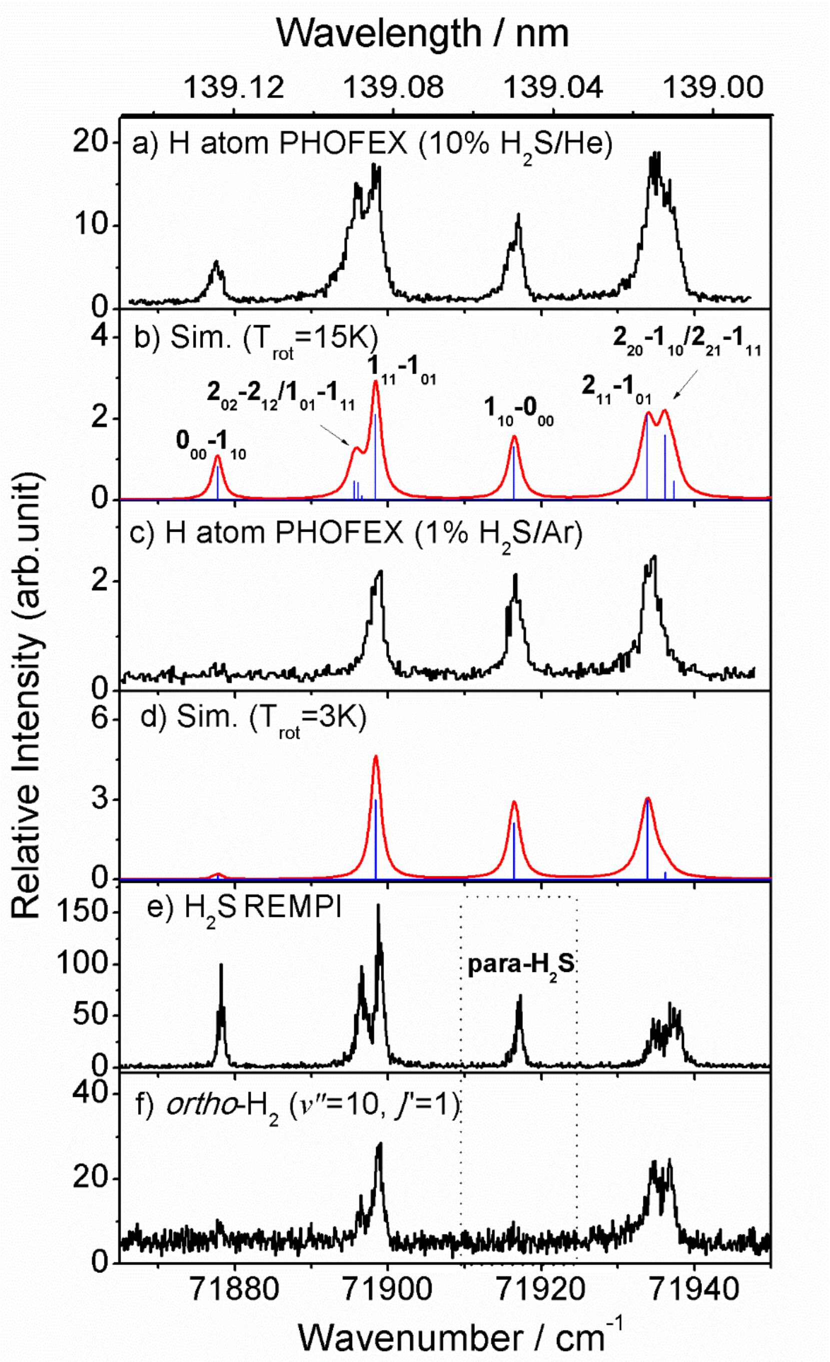

Supplementary Figure 2. Various $H_2S(^1B_1–X^1A_1)$ absorption/excitation spectra. H atom PHOFEX spectra, recorded using jet-cooled (a) 10% H_2S in He and (c) 1%H_2S in Ar gas mixtures. Panels (b) and (d) show the complementary PGOPHER simulations of the parent absorption spectrum, using spectroscopic parameters from refs. 2 and 3, the previously reported line broadening model outlined below and assuming T_{ns} = 300 K and T_{rot} = (b) 15 K and (d) 3 K. Panels (e) and (f) show, respectively, a 1 + 1′ parent REMPI spectrum recorded using a 10%H_2S in He gas mixture obtained by scanning the pump wavelength at λ ~139 nm and probing (by ionizing) with 532 nm photons and a PHOFEX spectrum obtained by probing

ortho-H_2 (v'' = 10, J'' = 1) fragments while scanning the pump wavelength around λ ~139 nm. The PGOPHER simulation of (e) returns T_{rot} ~20 K, while (f) shows parent resonances attributable to *ortho*-H_2S only and provides further demonstration of the conservation of nuclear spin symmetry in the $H_2S \rightarrow H_2 + S(^1D)$ dissociation process.

The line broadening model used in the PGOPHER simulations was originally developed to reproduce the envelope of the room temperature 3 + 1 REMPI spectrum of this $^1B_1$-X^1A_1 band.[2,5] Briefly, the width of an individual rovibronic transition in the present experiments is determined by the (level dependent) excited state lifetime and modelled as the sum of homogeneous and heterogeneous contributions using the function $\omega = \omega_0 + \omega'\langle J_b^2\rangle$ with, in the present case, ω_0 = 1.5 cm^{-1} and ω' = 0.15 cm^{-1}. The peak amplitude is reduced in a compensatory manner to conserve the rovibronic transition linestrength.

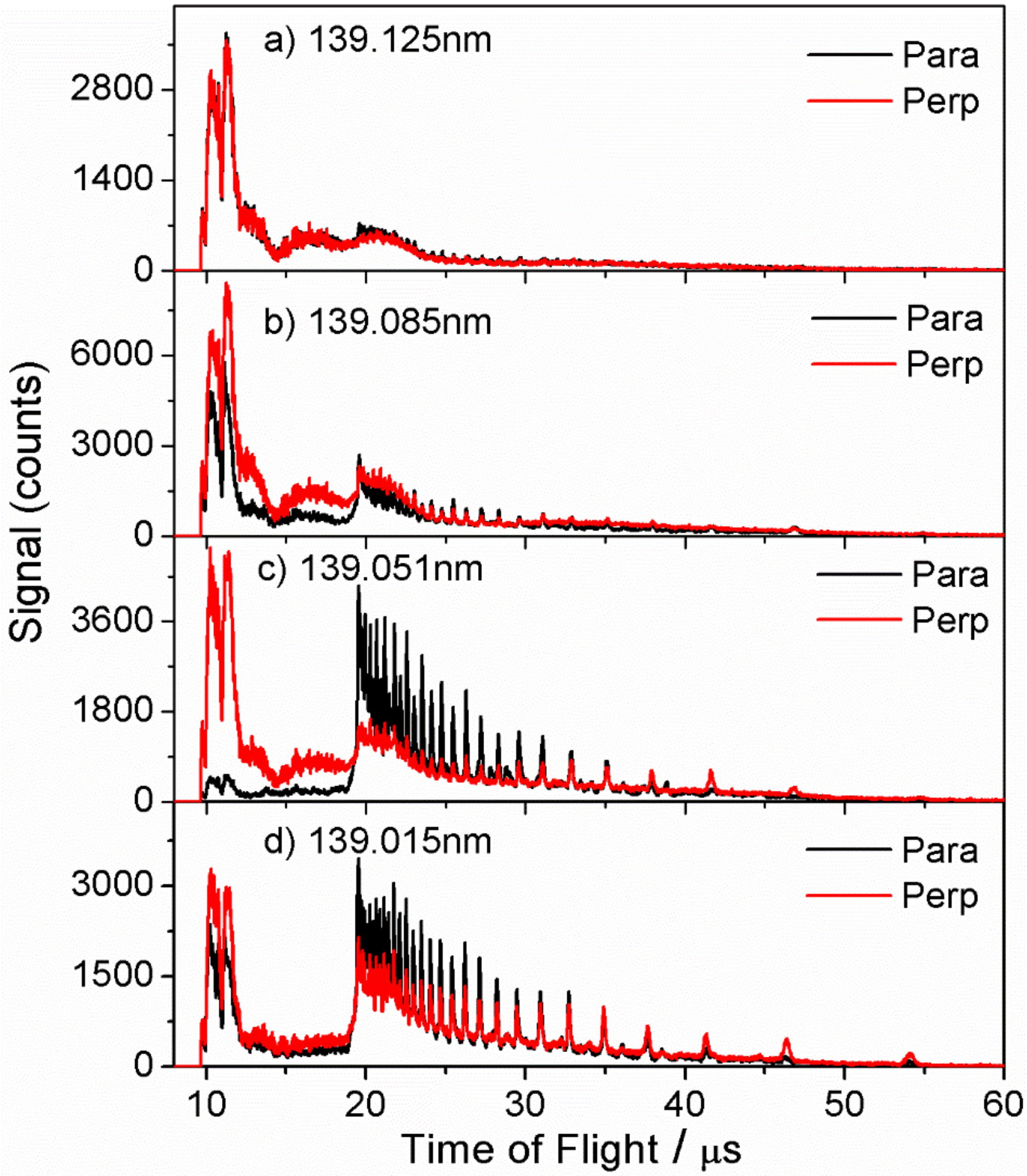

Supplementary Figure 3. H atom TOF spectra from H_2S photolysis at four wavelengths. H atom TOF spectra obtained following photolysis of a jet-cooled 30% H_2S in Ar sample at a) 139.125, (b) 139.085, (c) 139.051 and (d) 139.015 nm, with the polarization vector of the photolysis laser radiation ($\boldsymbol{\varepsilon}_{\mathbf{phot}}$) aligned, respectively, parallel (θ = 0°, shown in black) and perpendicular (θ = 90°, in red) to the detection axis.

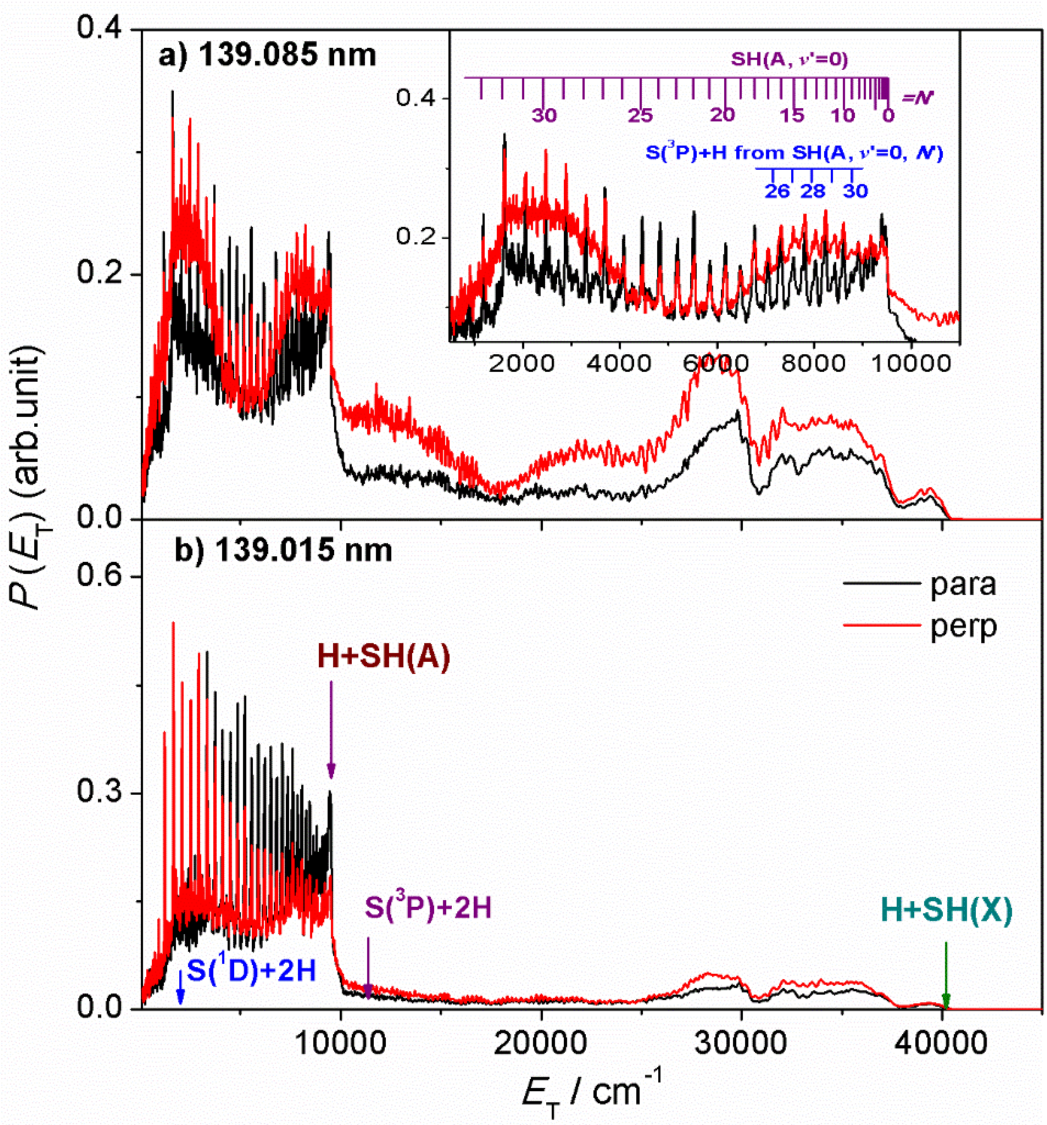

Supplementary Figure 4. The H + SH product translational energy (*P*(E_T)) spectra from H_2S photodissociation. *P*(E_T) spectra derived from H atom TOF spectra following photodissociation of H_2S at λ = (a) 139.085 and (b) 139.015 nm with $\boldsymbol{\varepsilon}_{\mathbf{phot}}$ aligned, respectively, parallel (θ = 0°, black) and perpendicular (θ = 90°, red) to the detection axis. The combs in the inset to panel (a) show the E_T values associated with formation of H atoms in conjunction with selected rovibrational levels of the primary SH(A) fragments and of the H atoms formed (along with S($^3P_2$) products from predissociation of primary SH(A, v' = 0) fragments in selected high N' levels. The maximum E_T values associated with each of channels I–IV are shown by vertical arrows.

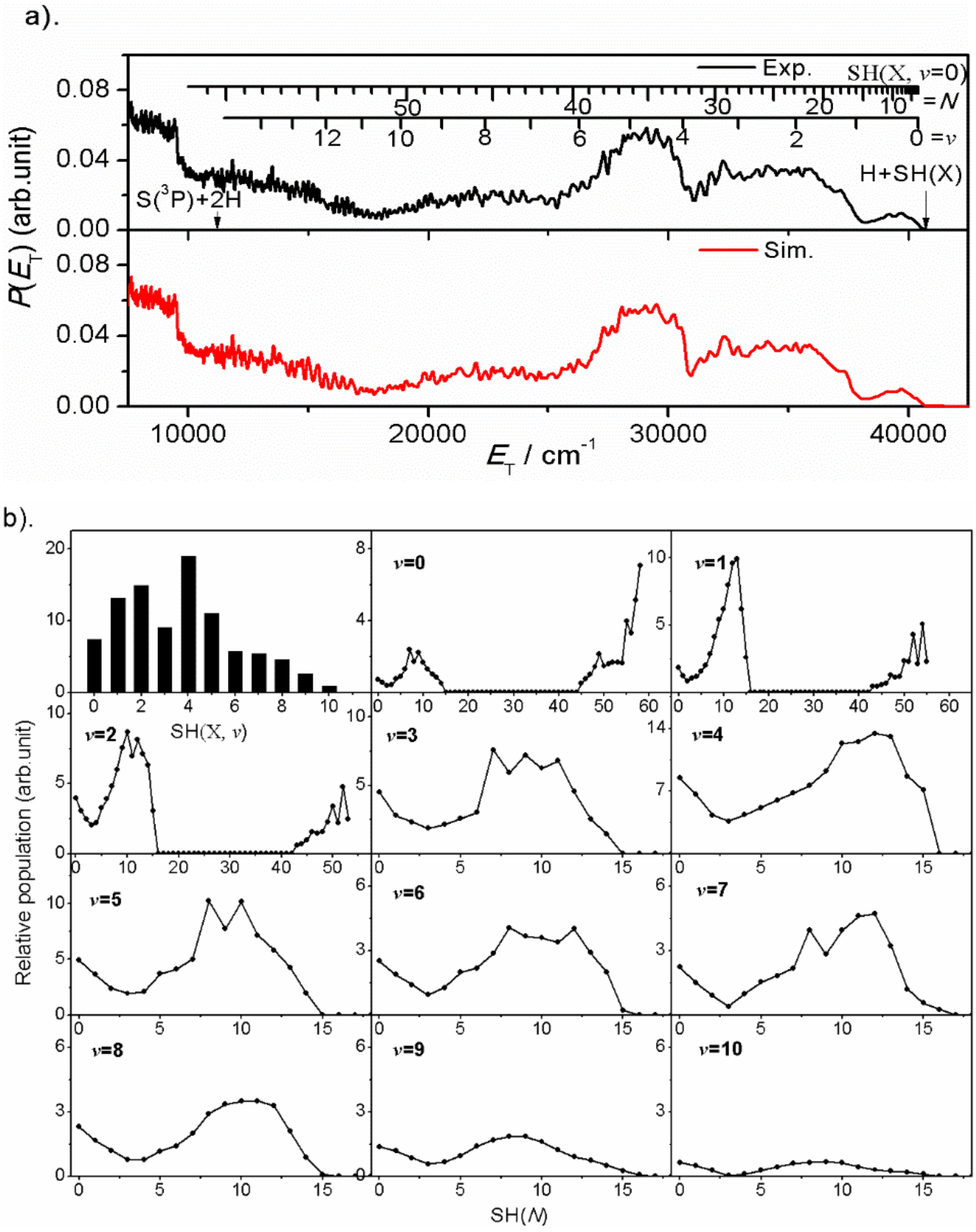

Supplementary Figure 5. The experimental and simulated translational energy spectra and the SH(X) product quantum state distributions. (a) $P(E_T)$ spectra from H atom TOF data recorded following excitation to the 0_{00} level at λ = 139.125 nm with $\boldsymbol{\varepsilon}_{\mathbf{phot}}$ aligned at the

90° angle to the detection axis (in black) and the best-fit simulation (below, in red) obtained using the SH(X, *v* and *N*) population distributions shown in panel (b).

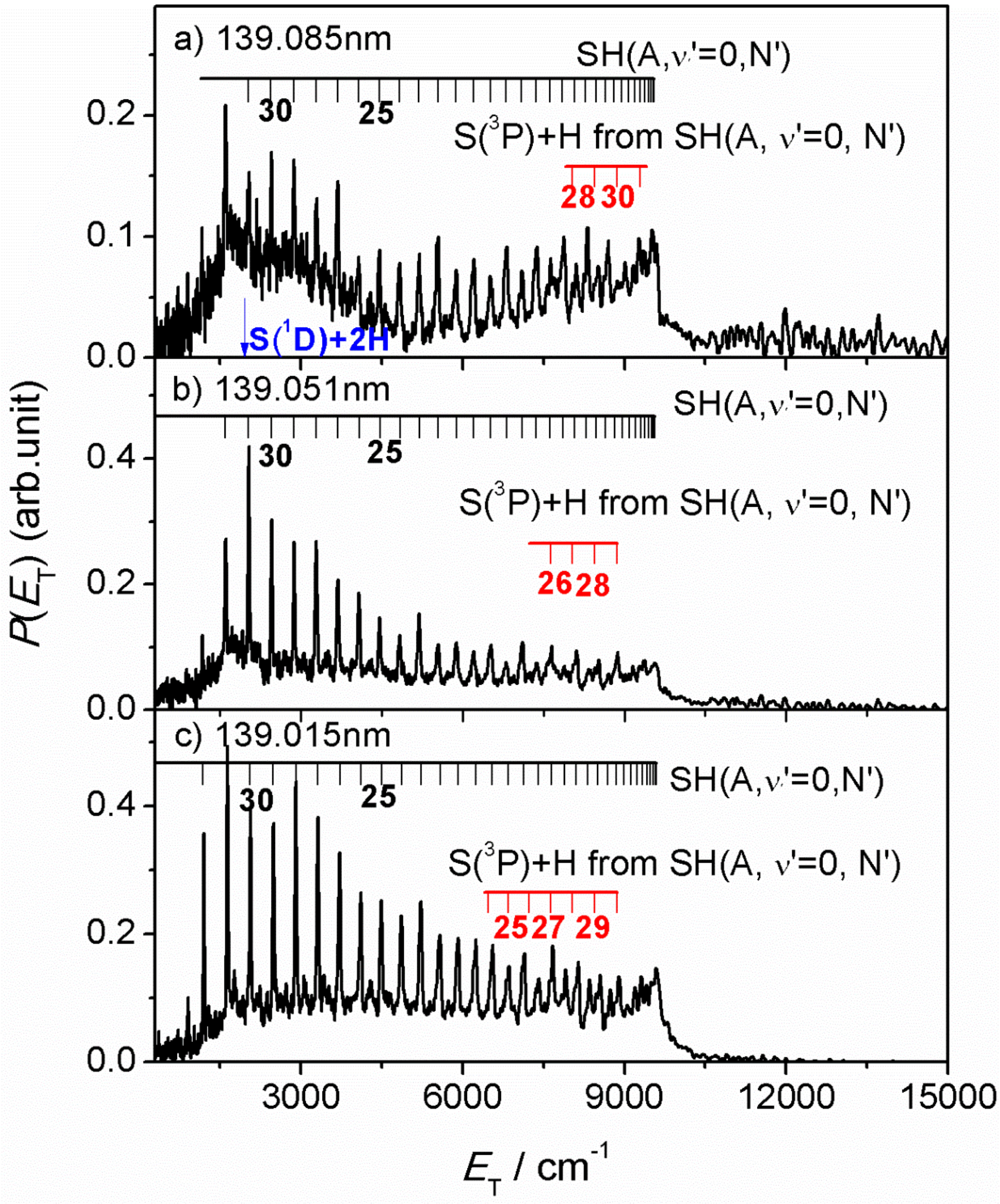

Supplementary Figure 6. The translational energy spectra highlighting the H+SH(A) product channel. $P(E_T)$ spectra highlighting the H + SH(A) product channel (process II) following excitation of a jet-cooled 30% H_2S in Ar sample at λ = 139.085, (b) 139.051 and (c) 139.015 nm with $\boldsymbol{\varepsilon}_{\mathbf{phot}}$ aligned at θ = 90°, after subtracting a suitably weighted amount of the $P(E_T)_{vib}$ distribution shown in Supplementary Fig. 5(a) to minimize the signal at E_T >10000 cm^{-1}. The superposed combs illustrate the expected E_T values of the H + SH(A, v' = 0, N') products and of the H + $S(^3P_2)$ products from predissociation of selected SH(A, v' = 0, N') levels.

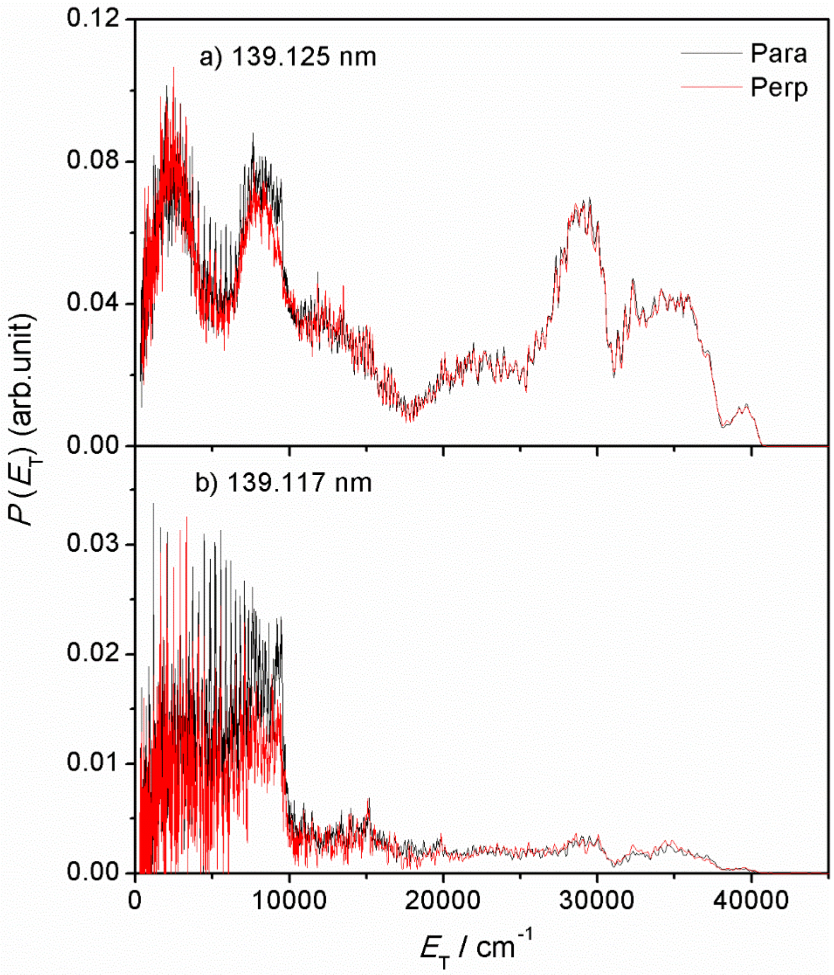

Supplementary Figure 7. The H+SH product translational energy spectra from H_2S photodissociation. $P(E_T)$ spectra from H atom TOF data recorded following excitation at λ = (a) 139.125 nm (on the peak of the 0_{00}-1_{10} line) and (b) 139.117 nm (exciting the background continuum), with $\boldsymbol{\varepsilon}_{\mathbf{phot}}$ aligned, respectively, parallel ($\theta = 0°$, black) and perpendicular ($\theta = 90°$, red) to the detection axis. Both show rotational structure attributable to formation of H + SH(A, v' and N') products.

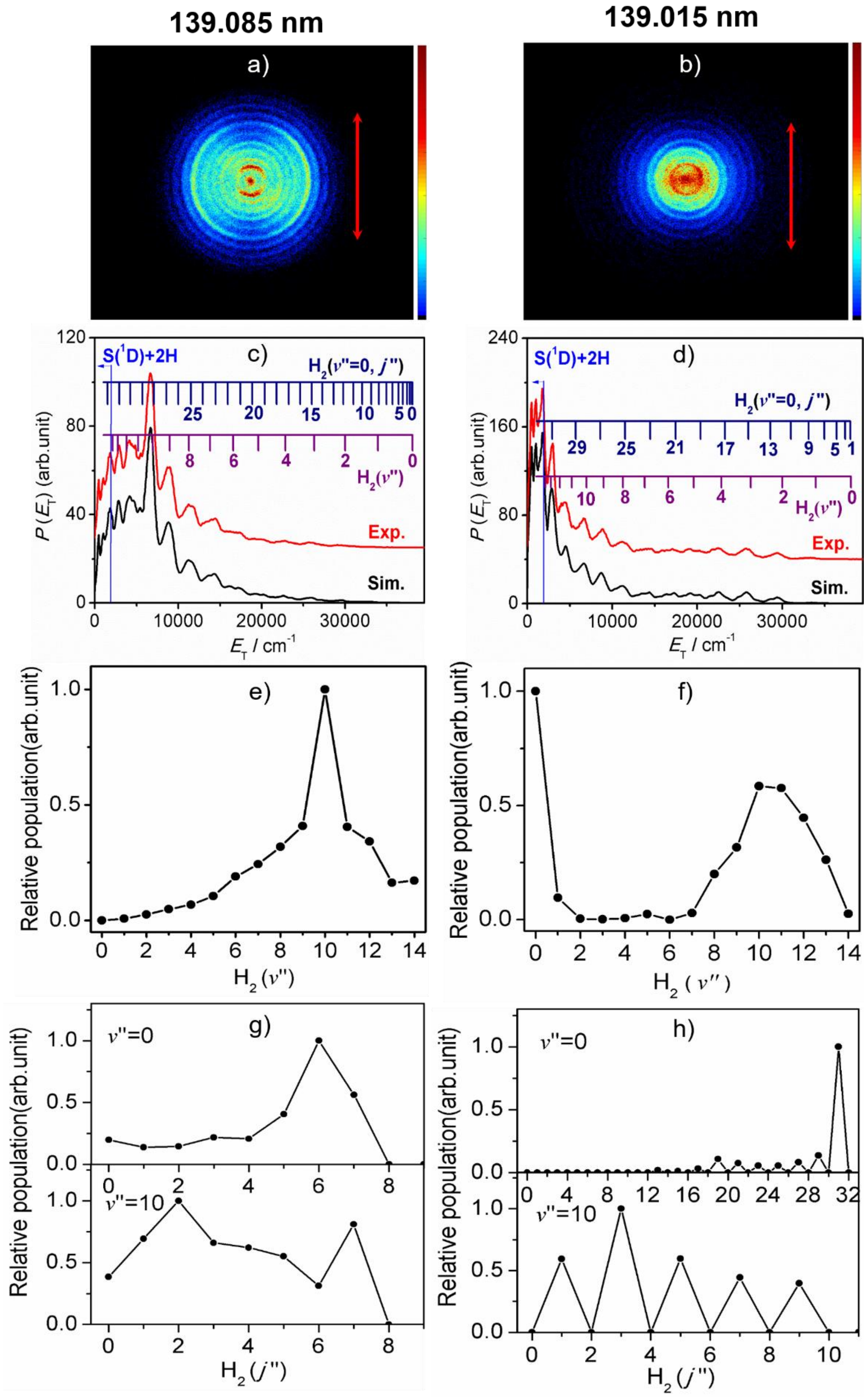

Supplementary Figure 8. The S(^{1}D) + H_2 product translational energy distributions and the H_2 product quantum state population distributions. Time-sliced velocity map images of the S($^1D_2$) photofragments from photolysis of H_2S at λ = (a) 139.085 and (b) 139.015 nm

with $\boldsymbol{\varepsilon}_{\mathbf{phot}}$ aligned vertically in the plane of the image (as illustrated by the doubled headed red arrow in (a)). The $P(E_T)$ spectra derived from these images are shown in (c) and (d), in red, along with the best-fit simulations of the spectra at E_T values down to the S(^{1}D) + H_2 dissociation threshold, in black and offset vertically for clarity. The superposed combs in (c) and (d) indicate the E_T values associated with formation of the various $H_2(v'', J'' = 0)$ and, respectively, the *ortho*- and *para*-J'' states of $H_2(v'' = 0)$. The energetic limit of the S(^{1}D) + 2H channel is marked by the vertical blue line. Panels (e) and (f) show the $H_2(v'')$ state population distributions returned by the respective best-fit simulations, while panels (g) and (h) show the J'' state population distributions for H_2 products in representative low ($v'' = 0$) and high ($v'' = 10$) vibrational states formed at each wavelength.

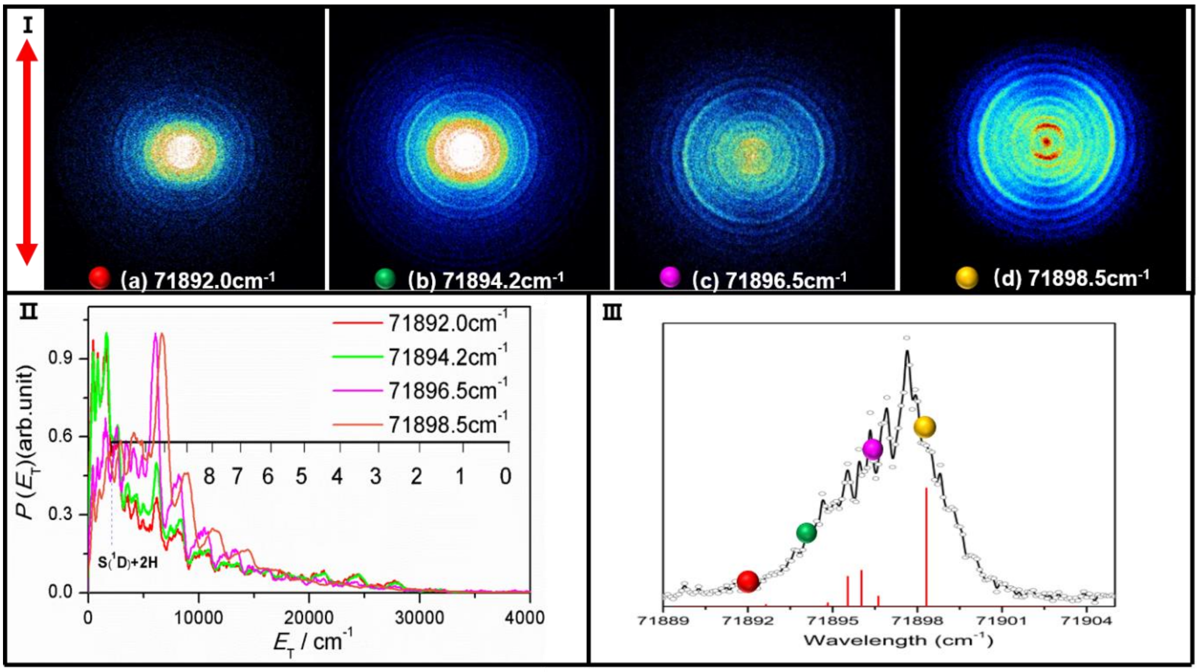

Supplementary Figure 9. The S(^{1}D) + H_2 product translational energy distributions with slightly different photolysis wavelengths. Time-sliced velocity map images of the S($^1D_2$) photofragments from photolysis of H_2S at (a) 71892.0 cm^{-1}, (b) 71894.2 cm^{-1}, (c) 71896.5 cm^{-1}, and (d) 71898.5 cm^{-1} with $\boldsymbol{\varepsilon}_{\mathbf{phot}}$ aligned vertically in the plane of the image (shown by the doubled headed red arrow in I). The $P(E_T)$ spectra derived from these images are shown in panel II, along with a superposed comb indicating the E_T values associated with formation of the various $H_2(v'', J'' = 0)$ products. Panel III shows the locations of these excitation wavenumbers along with the envelope of the blended feature at λ ~139.09 nm in the S(^{1}D) PHOFEX spectrum (reproduced from Fig. 1(b)).

The $P(E_T)$ distribution determined at 71896.5 cm^{-1} is similar to that at 71898.5 cm^{-1} (also shown in Supplementary Fig. 8(a)) indicating that, under the prevailing molecular beam conditions, the 1_{10}-1_{01} transition is still the dominant contributor to absorption at this wavenumber. But the $P(E_T)$ spectra obtained when making one (and two) additional ~2 cm^{-1} steps to lower wavenumber show obviously increased relative intensity and different structure at low E_T, implying an increased yield of H_2(low v'', high j'') products. As panel III shows, the dominant absorptions sampled at 71894.2 cm^{-1} access the 2_{02} and 1_{01} levels (both of which have $\langle J_b^2 \rangle > 0$). By 71892.0 cm^{-1}, most of the excitation is likely to be to the weak underlying continuum.

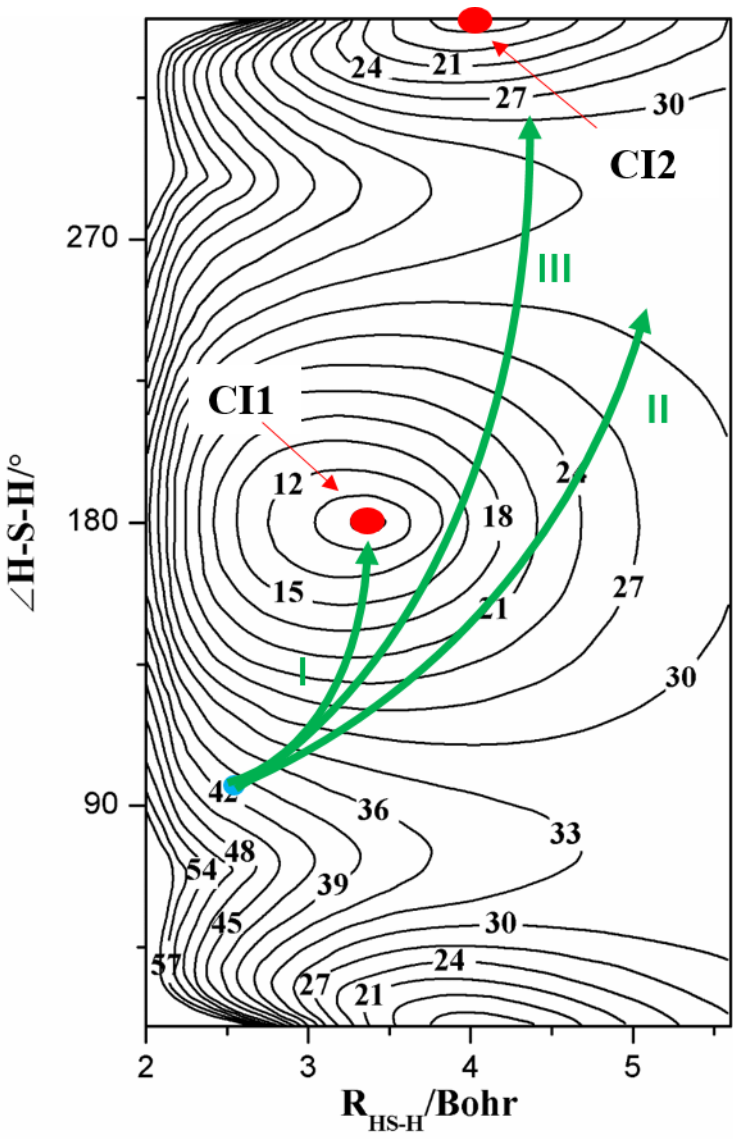

Supplementary Figure 10. The mechanism of H_2S photodissociation processes. Illustrative potential energy surface of the lowest excited $^1A'$ state of H_2S, plotted as functions of $R_{S–H}$ (the distance of one H atom departing from the S atom, with the other S–H bond held fixed at the ground state equilibrium bond length ($R_{S–H}$ = 2.5 bohr)) and the interbond angle ∠HSH (adapted from refs. 6 and 7). The contour labels are in units of 10^3 cm^{-1}, with the energy zero calculated at $R_{S–H}$ = 100 bohr (*i.e.* the H + SH(X) dissociation limit). The two regions of conical intersection with the $\tilde{X}$ state PES at linear H–S–H and S–H–H geometries (CI1 and CI2, respectively) are highlighted in red and the green curves depicting nuclear motions, originating from the equilibrium geometry of the $^1B_1$ state, that could lead to I: H + SH(X, low v'', high N''); II: H + SH(A, low v', high N') and III: H + SH(X) and S(^{1}D) + H_2 products. Note: the present experimental data shows no evidence for any contribution from possible process I.

References

[1] Western, C.M. PGOPHER, A Program for Simulating Rotational, Vibrational and Electronic Spectra, *J. Quant. Spectrosc. Rad. Trans*. **186**, 221-242 (2017).

[2] Ashfold, M.N.R. & Dixon, R.N. Multiphoton ionisation spectroscopy of H_2S: A reinvestigation of the $^1B_1$–$^1A_1$ band at 139.1 nm. *Chem. Phys. Letts*. **93**, 5-10 (1982).

[3] Morgan, W.J., Huang, X.C., Schaefer, H.F. & Lee, T.J. Astrophysical sulfur in diffuse and dark clouds: The fundamental vibrational frequencies and spectroscopic constants of hydrogen sulfide cation (H_2S^+) *MNRAS* **480**, 3483–3490 (2018).

[4] Lee, L.C., Wang, X. & Suto, M. Quantitative photoabsorption and fluorescence spectroscopy of H_2S and D_2S at 49-240 nm. *J. Chem. Phys*. **86**, 4353-4361 (1987).

[5] Mayhew, C.A., et al., High-resolution studies of the electronic spectra of H_2S and D_2S. *J. Chem. Soc. Faraday Trans. 2*, **83**, 417-434 (1987).

[6] Cook, P.A., Langford, S.R., Dixon, R.N. & Ashfold, M.N.R. An experimental and ab initio reinvestigation of the Lyman-α photodissociation of H_2S and D_2S. *J. Chem. Phys*. **114**, 1672-1684 (2001).

[7] Zhou. J., et al., Ultraviolet photolysis of H_2S and its implications for SH radical production in the interstellar medium. *Nat. Comm*. **11**, 1547 (2020).
